# Do Extreme Climate Events Cause the Degradation of *Malus sieversii* Forests in China?

**DOI:** 10.3389/fpls.2021.608211

**Published:** 2021-06-16

**Authors:** Qianjuan Shan, Hongbo Ling, Hangzheng Zhao, Mengyi Li, Zikang Wang, Guangpeng Zhang

**Affiliations:** ^1^State Key Laboratory of Desert and Oasis Ecology, Xinjiang Institute of Ecology and Geography, Chinese Academy of Sciences, Urumqi, China; ^2^University of Chinese Academy of Sciences, Beijing, China; ^3^Xinjiang Aksu Oasis Agro-Ecosystem Observation and Experiment Station, Urumqi, China; ^4^School of Chemical and Environmental Engineering, China University of Mining and Technology, Beijing, China; ^5^School of Civil Engineering, Tianjin University, Tianjin, China

**Keywords:** extreme climate indices, atmospheric circulation, anthropogenic impact, dendrochronology of *Malus sieversii* trees, the degraded wild fruit forest

## Abstract

Frequent extreme climate events have attracted considerable attention around the world. *Malus sieversii* in Xinjiang is the ancestor of cultivated apple, and it is mainly distributed in the Ili river valley at end of the Tianshan Mountains. Wild fruit forests have been degraded, but the cause remains unclear. In order to identify whether extreme climate events caused this degradation reanalysis data and atmospheric circulation indices were used to determine the trends and the reasons for extreme climate changes. Subsequently, we further investigated the effect of extreme climate events on wild fruit forest using characteristics of extreme climate indices and tree-ring chronology. We found increasing trends in both extreme precipitation and warm indices, and decreasing trends in cool indices. Extreme climate events were mainly associated with the Atlantic Multidecadal Oscillation (AMO). Analysis of data of wind and geopotential height field at 500 hPa showed that strengthening wind, increasing geopotential height, cyclone and anti-cyclone circulation drivers contributed to extreme climate events. In the non-degraded region, there were significant positive correlations between tree-ring chronology and both extreme precipitation and extreme warm indices (except for warm spell duration indicator). The other extreme indices (except for heavy rain days) had a large correlation range with tree-rings in a 4–8-year period. These results indicated that extreme precipitation and extreme warm indices intensified *M. sieversii* growth of the non-degraded region on multi-time scales. In contrast, the degraded region showed insignificant negative relationship between tree-ring chronology and both extreme precipitation and extreme warm indices [except for warm spell duration index (WSDI)], and significant negative correlations in a 4–8-year period were detected between tree-ring chronology and most of the extreme precipitation indices, including heavy rain days, very wet days, cold spell duration indicator, simple precipitation intensity index (SDII), and annual total precipitation. Under the long disturbance of inappropriate anthropic activities, extreme climate has caused the outbreak of pests and diseases resulting in the degeneration of wild fruit forest. Our study provides scientific guidance for the ecosystem conservation in wild fruit forest in China, and also across the region.

## Introduction

Global warming has led to increasing of extreme climate events around the world in the past 60 years, such as heat waves, high rainfall and flooding (Powell and Keim, 2015; Sun et al., 2016; Xiao et al., 2016). Extreme climate events have large impact on natural ecosystems and human society (Mullan et al., 2012; Song et al., 2015; Sun et al., 2016), and have characteristics of suddenness, unpredictability and destructiveness (Alexander et al., 2013; Raineri, 2013). Therefore, the majority of researchers suggest that future study of climate change should focus on extreme rather than mean climate change (Shi et al., 2018). Arid and semi-arid areas in northwest China are among the most sensitive areas to climate change, due to vast desert basins, high mountains and being far from the sea (Shi et al., 2007). Extreme weather events occur frequently throughout the arid region of northwest China, such as extreme heat, drought and snowstorms (Li et al., 2019; Han et al., 2020; Pi et al., 2020; Xie et al., 2020). Extreme temperatures are clearly associated with atmospheric circulation, and extreme precipitation shows spatial differences (Wang B. L. et al., 2013; Deng et al., 2014; Han et al., 2016). Notably, the Ili river valley at the western end of the Tianshan Mountains, the world-famous distribution area of wild fruit forest, is not only the vital west water vapor channel in northwest China, but also is the transitional zone of westerly circulation and monsoons (Qian et al., 2001; Shi et al., 2007), which results in frequent extreme weather events there. However, the change characteristics of the extreme climate and its driving mechanism have not been explored, therefore, the first aim in this study is to solve the above scientific problem.

The impact of extreme climate on forest ecosystems has both positive and negative aspects according to previous studies. The negative effects of extreme climate are following expressed by drought, heat waves and wildlife and insect disturbance causing increased tree mortality and dead wood, reduced species diversity and reduced productivity (Ciais et al., 2005; Rammig et al., 2010; Birch, 2014). Extreme high temperature causes lower water use efficiency by accelerating the transpiration of vegetation, and also exacerbates outbreaks of forest pests and diseases (Kolb et al., 2016; Pettit et al., 2020), all of which slow tree growth. Extreme drought results in reduced photosynthesis, which decreases growth and increases mortality of trees (Feldpausch et al., 2016), and also impacts on phenology, such as flowering cycles and end-of-season vegetative growth (Nagy et al., 2013; Cardil et al., 2014). Extreme low temperature such as frost and freezing can impair the extension of leaves (Vanoni et al., 2016), thus reducing photosynthesis and carbon absorption and so slowing tree growth. In contrast, the positive effects of extreme climate follow. Global warming can exacerbate the hydrological cycle leading to changes in rainfall patterns (both spatially and temporally) with extreme wetness and drought becoming more frequent (Min et al., 2011; Cook et al., 2015; Touma et al., 2015; Huang et al., 2016). Extreme precipitation not only satisfies the basic physiological and biochemical processes of vegetation, but also restrains the negative impact of drought on trees caused by high temperature. Compared to extreme drought, extreme wetness compensates for post-drought carbon loss in dry forests, and so enhances tree growth (Jiang et al., 2019). The above extreme climate changes have a significant impact on forest composition and forest main productivity. Therefore, the study of extreme climate change has become an important ecological problem to understand changes in forest growth (Stocker et al., 2013). *Malus sieversii* forest in Xinjiang is one of the major original areas of wild fruit trees worldwide, and includes many wild resources. These include the dominant species of *M. sieversii* confirmed as the ancestor of cultivated apple (Duan et al., 2017) and which has become a Grade II protected plant in China (Archetti, 2009). Following the increasing mortality of *M. sieversii*, the wild fruit forest ecosystem has been seriously damaged and some wild fruit forest region degraded obviously (Fang et al., 2019). One study showed that *M. sieversii* growth was associated with spring precipitation and winter temperature (Panyushkina et al., 2017). However, whether extreme climate change has positive or negative effects on this forest growth has not been determined, and we wonder if the degradation of wild fruit forest may be caused by extreme change, these scientific problems are addressed in this study.

In investigating the above two scientific issues, daily temperature and precipitation data of three meteorological stations were used to calculate extreme climate indices. Identifying the change characteristics of extreme climate and its driving mechanism can be determined using the relationships between extreme climate indices and atmospheric circulation indices. Among many kinds of paleoclimatic substitute data, tree-rings offer a great advantage in research on paleoclimatic information archives with the benefits of accurate annual resolution, a large number of replications, and easy access, long-scale hydrological and climate change chronologies obtained from tree-ring data have unparalleled advantages and great potential in this respect (Fan et al., 2020). Moreover, tree-rings are a sensitive material to reflect the situation of tree growth, and can also record the historic climate and surrounding change (Fritts, 1976). Tree-ring width chronology has been used to reflect the tree growth and the response to changes of surrounding in many studies. Decuyper et al. (2020) indicated that the years of extreme climate (drought and high temperature) resulted in a matching small tree-ring chronology index. In contrast, the tree-ring chronology index is large with the occurrence of extreme wetness (Jiang et al., 2019). Additionally, tree-ring width chronology can also record the response to historic anthropogenic changes (Andrade et al., 2019; Mazza et al., 2020). Human disturbance such as deforestation, pruning and grazing decrease tree growth by reducing photosynthesis, and the resulting tree-ring chronology index is small. In view of the advantages of tree-ring technology, we use it to analyze the change of tree growth, in two regions. We contrastively analyzed the tree-ring chronology of *M. sieversii* in the wild fruit forests in Gongliu County (a non-degraded region) and Xinyuan County (a degraded region) in the Ili river valley (see Supplementary Table 1 for the classification of degradation degrees), where wild fruit forests are widely distributed. The influence process of extreme climate change on the growth of wild fruit forests was revealed by comparing and analyzing the correlations between extreme climate indices and tree-ring chronology. The reasons for the degradation of wild fruit forests in Xinjiang, including both extreme climate and human activities, were explored.

## Materials and Methods

### Study Site

**FIGURE 1:**
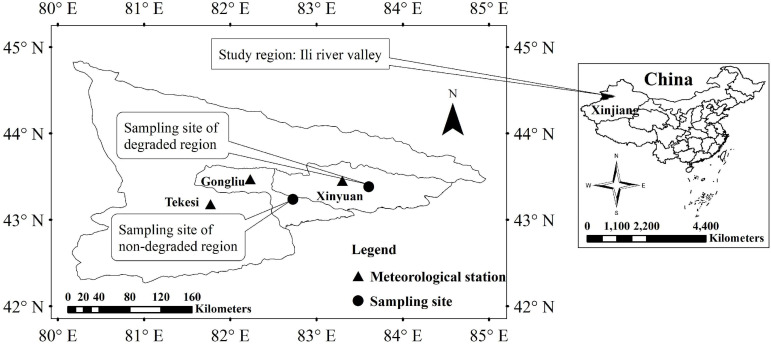
Overview of the study area. The vector map of China was obtained by vectorizing the Chinese map [Approval Number: GS (2016) No. 2893] supervised by the Ministry of Land and Resources of China.

*Malus sieversii* forest in Xinjiang China is the origin of the world’s cultivated apples (Wang et al., 2018), and *M. sieversii* is the dominant species in the wild fruit forest mainly distributed on the Tianshan Mountains on both sides of the Ili river valley, China, with a total area of 8,786 km^2^. The longitude and latitude ranges are 80°42′52″–83°37′17″E and 43°13′14″–44°26′28″N, and the height above sea level is 1,100–1,700 m. The climate in the study area is a typical temperate continental climate. The water vapor transport in summer and winter comes from the Arctic and Atlantic Oceans, respectively. The average annual precipitation in this area is 260–800 mm, and average annual temperature is 10.4°C (Wang H. et al., 2013; Kong et al., 2017). Compared with cultivated apple, *M. sieversii* has a wider range of adaptation to drought, extreme temperature, and pathogens (Luby et al., 2001; Dzhangaliev, 2002). In order to determine the causes of degradation of wild fruit forests, we selected two wild fruit forest sample points (with similar altitudes of 1,100–1,500 m): in Gongliu County (a non-degraded area) with less human interference, the dead trees represent less than one third of the total trees, and the range of canopy loss is 10–40% in Xinyuan County (a degraded area) with greater human interference, 1/3–3/4 of all trees are dead, and the tree canopy loss exceeds 40%. The soils in the two sampling sites are mainly mountain black brown soils (Lin and Cui, 2000), and the species distributions at the two sampling points were similar (up to 441 species). The distribution of sampling sites in the study area is shown in Figure 1.

### Data Source

(1) Tree-ring data: Tree-ring width can reflect the radial growth of individual trees, during the vegetation season. In order to avoid being affected by competition, 94 wild apple trees (49 trees in the degraded area, 45 trees in the non-degraded area) were selected from isolated, mature and healthy trees in the two regions, In this way, the selected trees were less affected by the competition of surrounding plants and the growth was more steady. Then, we used Pressler increment borers to extract two radial cores per tree, which were brought back to the laboratory in hollow plastic tubes. (2) Climate data: This study used the daily temperature and precipitation data of meteorological stations including Gongliu, Xinyuan, and Tekesi for calculating the extreme indices for 1961–2017,during which time these stations were not relocated. The water vapor of northwest China is mainly from moisture brought by the westerlies (Chen et al., 2017). To investigate the oceanic influence on the region climate change, we selected four main atmospheric circulation indices including Atlantic Multidecadal Oscillation index (AMO), Atlantic Meridional Mode index (AMM), Pacific Decadal Oscillation index (PDO) and North Atlantic Oscillation index (NAO)^1^, which are the main oceanic influence of the climate change in northwest China (Chen et al., 2014, 2017; Steinman et al., 2015; Sun et al., 2020). To quantify the reasons for changes in large-scale atmospheric circulation, monthly mean geopotential height and wind fields at 500 hPa during 1961–2017 were derived from the NCEP/NCAR reanalysis data^2^, and the resolution of the reanalysis variables was is 2.5° × 2.5°.

### Method

**FIGURE 2:**
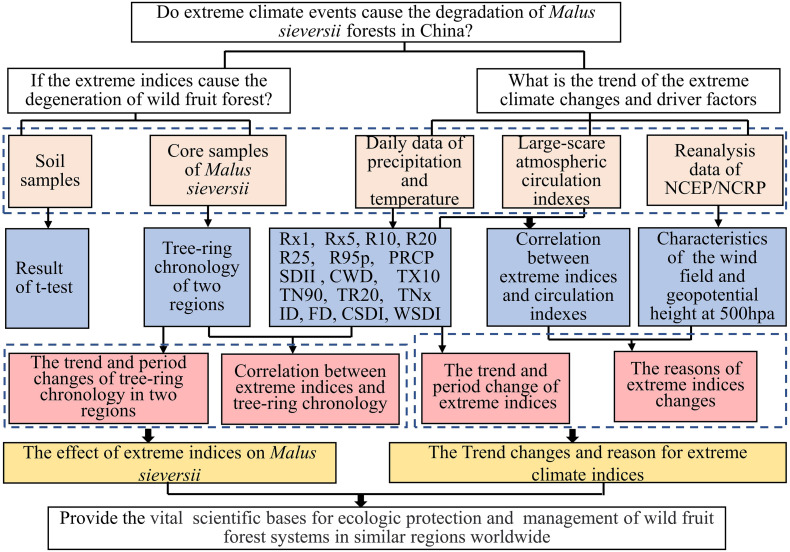
Flow chart of the study.

In order to determine whether soil conditions contribute to the difference in tree growth between the two areas, we used *t*-tests in Statistical Product and Service Solutions software (SPSS, IBM Inc., Armonk, NY, United States) to test the two regions soil differences in physical and chemical properties of soil of the two regions. Then, we calculated the required extreme climate indices using climate data and developed the tree-ring width chronologies of the two regions from the tree core samples. Moreover, characteristic analysis was applied to *M. sieversii* tree-ring chronology and the extreme climate indices series using Mann-Kendall trend tests, Mann–Whitney *U* tests and periodic analysis. Meanwhile, based on the potential impact of climate change in northwest China, we performed Pearson’s correlation analysis between the atmospheric circulation indices and extreme climate indices to explore the impact of atmospheric circulation types on extreme climate changes in the study area. Finally, through Pearson’s correlation and wavelet coherence analysis of extreme climate indices and tree-ring chronology, we discussed the multi-scale effects of extreme climate events on tree growth in the study area, and analyzed the main reasons for degradation of wild apple forest using the data form the *M. sieversii* forests. The specific flow is shown in Figure 2, with the method given in the following.

#### Sample Processing and Chronology Development

After sampling, core samples were air-dried, before ring-width measurements, all samples were sanded with progressively finer grades of sandpaper (120–1,200) to make the growth ring boundaries more visible. Ring widths were measured with a LINTAB 6 measuring system (Rinntech-Metriwerk GmbH & Co. KG, Heidelberg, Germany) at a precision of 0.01 mm using the tree-ring analysis software TSAP-Win (Rinn, 2003). The COFECHA cross-dating quality control program was used for cross-dating test and calculating moving correlations between each individual series and the mean site series (Holmes, 1983); some cores with small correlation coefficients, short tree age and many singular points were eliminated. In order to get a long time-series, 12 long-core samples to establish the chronology in the non-degraded region (common period: 1955–2017), and 20 core samples in the degraded region (common period: 1965–2017) were used to establish the dendrochronology. The ARSTAN program (calculation of tree-ring chronology) was used to establish the tree-ring chronology of the two study areas on the basis of the accurate cross-dating (Cook, 1985). The growth trend of the tree-ring chronology was fitted using a 25-year step spline function and the tree-ring chronology indices were calculated by dividing the measured value by the fitted value of tree ring width. The chronology statistics are given in Supplementary Table 3.

#### Classification and Definition of Extreme Indices

In this study, we calculated all extreme indices based on mean daily temperature and precipitation data of three stations. Data quality control was performed using RClimDex software^3^, which was developed and maintained by Zhang and Yang at the Climate Research Branch of the Meteorological Service of Canada. We selected 17 extreme indices from 27 core indices introduced by the Expert Team on Climate Change Detection and Indices (ETCCDI)^4^ (You et al., 2013; Guan et al., 2015; Rahimi and Hejabi, 2018). We grouped the extreme temperature indices into four categories (Choi et al., 2009; Wang et al., 2013). The first category was the indices based on the relative (floating) threshold, abbreviated as the relative indices, we selected the cold days (TX10p: number of days when daily max temperature < 1961–2017 10th percentile) and warm nights (TN90p: number of days when daily min temperature > 1961–2017 10th percentile). The second category was indices based on original observation data and fixed thresholds, abbreviated as the absolute indices, we selected the numbers of hot nights, icing days, and frost days (TR, ID, and FD, respectively). The third category was the highest and lowest daily maximum and minimum temperatures for the month, abbreviated as the extreme value indices, we selected the monthly maximum value of daily minimum temperature (TNx). The fourth category was other indices, we selected the WSDI and cold spell duration index (CSDI).

Bonsal’s nonparametric scheme was used to determine the threshold of extreme precipitation (Bonsal et al., 2010), the specific calculation follows:

If the number of one climate factor is *N*, putting these values in ascending order x_1_, *x*_2_,…,*x_m_*,…,*x_n_*, and the probability that a value is less than or equal to *x*_*m*_:

P=(m-0.31)/(n+0.38)

where *P* is the percentile, *m* is the sequence number of *x*_*m*_, and *n* is the number of climate factors.

The percentile threshold method was using to calculate R95 (annual total precipitation when daily total precipitation > 95th percentile). Eight extreme precipitation indices were all calculated by RClimDex software: maximum 1-day precipitation (RX1), maximum 5-day precipitation (RX5), annual count of days when annual total precipitation ≥10, 20, and 25 mm (R10, R20, and R25, respectively), annual total precipitation (PRCP), simple precipitation intensity index (SDII) and consecutive wet days (CWD).

#### Nonparametric Tests

##### Mann-Kendall trend test

The Mann-Kendall statistical test is a nonparametric test method. The time series (*X*_1_, *X*_2_, *X*_3_, …, *X*_*n*_) were compared in turn, and the results were recorded as sgn (θ):

(1) $$sgn\left(\theta \right)=\left\{ \begin{array}{c} 1,\theta >0\\ 0,\theta =0\\ -1,\theta <0 \end{array}\right.$$

The Mann-Kendall statistical calculation result was:

(2) $$s=\sum_{i=1}^{n}\sum_{k=i+1}^{n}sgn\left(x_{k}-x_{i}\right)$$

where *x*_*k*_ and *x*_*i*_ are random variables and n is the length of the selected data sequence.

The test statistic *Z*_*c*_ was calculated as follows:

(3) $$Z_{c}=\left\{ \begin{array}{c} \frac{s-1}{\sqrt{var\left(s\right)}},s>0\\ 0,s=0\\ \frac{s+1}{\sqrt{var\left(s\right)}},s<0 \end{array}\right.$$

In this equation, | *Z*_*c*_| ≥ 1.96 and | *Z*_*c*_| ≥ 2.58 indicated that the sample sequence had a significant trend change at *p* < 0.05 and 0.01, respectively, *Z*_*c*_ > 0 indicated a rising trend and *Z*_*c*_ < 0 indicated a declining trend (Ling et al., 2014).

##### Mann–Whitney *U* test

The abrupt-change point should be determined before the abrupt-change test. In this paper, the abrupt-change point was determined according to the trend change of the cumulative anomaly value.

The basic principle of Mann-Kendall test follows:

Suppose the time series is *X* = (*X*_1_, *X*_2_, …, *X*_*n*_) and its sub-sequence *Y* = (*X*_1_, *X*_2_, …, *X*_*n*_), and *Z* = (*X*_*n*1+1_, *X*_*n*1+2_, …, *X*_*n*1+*n*2_). The abrupt-change test is calculated as:

(4) $$Z_{c}=\frac{\sum_{t=1}^{n_{1}}r\left(x_{t}\right)-n_{1}\left(n_{1}+n_{2}+1\right)/2}{\sqrt{n_{1}n_{2}\left(n_{1}+n_{2}+1\right)/12}}$$

In this equation, *r* (*x*_*t*_) is the rank of the observed value, *n*_1_ is the number of time series before the abrupt change and *n*_2_ is the number of time series after the abrupt change, then *n*_1_ + *n*_2_ = *n*. If –*Z*_1–*a*/2_ ≤ *Z*_*c*_ ≤ *Z*_1–*a*/2_, then the null hypothesis is accepted. Given the test level of a, *Z*_1–*a*/2_ is the quantile of the standard normal distribution of 1 – *a*/2 (Ling et al., 2014).

#### Analysis of Wavelet Coherence

The wavelet variance represents the strength (energy) of cyclical fluctuation of time series in this scale, and the scale at the corresponding peak value is the main period of this series (Gao and Li, 1993). Therefore, the periodic changes of the climate factors and the tree-ring chronology change were analyzed using wavelet variance.

The wavelet variance is:

(5) $$W_{p}\left(a\right)=W_{f}{\left(a,b\right)}^{2}$$

Wavelet coherence spectrum is used to measure the local correlation degree of two time series in time-frequency space. The wavelet coherence spectrum of two time series *X* and *Y* is defined as:

(6) $$R_{n}^{2}\left(s\right)=\frac{{\left|S\left(s^{-1}W_{n}^{XY}\left(s\right)\right)\right|}^{2}}{S\left(s^{-1}{\left|W_{n}^{X}\left(s\right)\right|}^{2}\right)\cdot S\left(s^{-1}{\left|W_{n}^{Y}\left(s\right)\right|}^{2}\right)}$$

(7) $$S\left(W\right)=S_{scale}\left(S_{time}\left(W_{n}\left(s\right)\right)\right)$$

*S*_*scale*_ represents smoothing along the wavelet scaling axis and *S*_*time*_ represents smoothing along the wavelet time translation axis.

(8) Stime(W)|=s(Wn(s)*c1-t2/2s2)|sSscale(W)|=n(Wn(s)*c2∏(0.6s))|n

In this equation, *c*_1_ and *c*_2_ are standardized constants, Π is the rectangular function and the parameter 0.6 is a scale determined based on experience and is related to the solution of the Morlet wavelet wavelength. The significance test of wavelet coherence spectrum adopts the Monte Carlo method. The 95% confidence interval given in this paper is for the wavelet coherence spectrum, and only the phase difference arrow of $R_{n}^{2}\left(s\right)\geq 0.5$ is marked in the wavelet coherence spectrum.

## Results

### Extreme Climate Indices

#### Trend and Periodic Variation of Extreme Precipitation Indices

**FIGURE 3:**
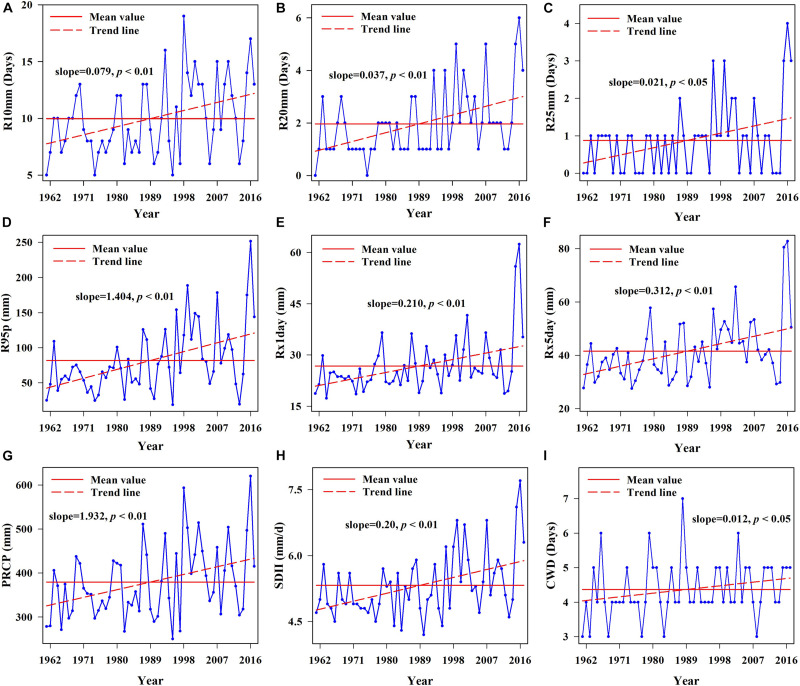
Linear trend charts of annual extreme precipitation indices during 1961–2017. **(A)** Linear trend chart of light rain days, **(B)** linear trend chart of moderate rain days, **(C)** linear trend chart of heavy rain days, **(D)** linear trend chart of very wet days, **(E)** linear trend chart of maximum one-day precipitation, **(F)** linear trend chart of maximum five-day precipitation, **(G)** linear trend chart of annual total precipitation, **(H)** linear trend chart of simple precipitation intensity index, and **(I)** linear trend chart of consecutive wet days.

The extreme precipitation indices for 1961–2017 (Figure 3) showed significantly increasing trends (*p* < 0.05) for R25 and CWD, and the other extreme precipitation indices showed very significant increasing trends (*p* < 0.01) according to Mann-Kendall trend tests. The number of days of moderate rain, heavy rain and rainfall increased change rates of 0.79, 0.37, and 0.21 d/10 years, respectively (Figures 3A–C). Heavy rainfall (R95p) increased at a rate of 14.04 mm/10 years, and heavy rainfall was higher than the average in most years after 1989 (Figure 3D). The maximum 1- and 5-d precipitation (R × 1 day, R × 5 day) increased at a rate of 0.21 and 3.12 mm/10 years, respectively. The increase extent was large after 2002 and 2003 (Figures 3E,F). The annual total precipitation/snow amount (PRCP)increased at a rate of 19.32 mm/10 years (Figure 3G), precipitation intensity (SDII) increased at 2.0 mm/d/10 years (Figure 3H) and CWD increased at 0.12 d/10 years, with the fluctuations relatively stable after 1989 (Figure 3I). In general, the precipitation intensity was weak before the 1980s, precipitation began to increase in the 1980s and continued to increase after the 1990s, precipitation in the study area showed a continuous increasing trend. The period and abrupt-changes test results showed (Table 1) that the R10 and PRCP indices had a very significant increasing abrupt-changes in 1997, CWD had significant increasing abrupt-changes in 1978 and the remaining precipitation indices had very significant increasing abrupt-changes in 1995. From the perspective of wavelet variance, the common period was 13 years, and the period was not significant. Overall, after the 1990s, rainfall in the study area increased significantly.

**TABLE 1:** Results of abrupt-change test and period of all extreme indices.

Extreme precipitation	Abrupt-year	|*Zc*|	H0	Period	Extreme temperature	Abrupt-year	|*Zc*|	H0	Period
R10 mm	1997	3.49	R	7,12,21	TN90	1996	6.25	R	16,21,25
R20 mm	1995	3.66	R	9,12,21	TNx	1995	5.20	R	9,16,21
R25 mm	1995	2.52	R	8,12,21	TR20	1996	4.75	R	16,21,25
R95p	1995	3.65	R	9,13,21	WSDI	2000	2.99	R	6,14,21
Rx1	1995	2.71	R	9,13,21	FD	2000	4.92	R	16,21,25
Rx5	1995	3.57	R	6,13,21	ID	1994	2.66	R	14,21,25
PRCP	1997	3.14	R	7,13,21	TX10	1994	4.12	R	12,21,25
SDII	1995	3.56	R	9,13,21	CSDI	1988	4.49	R	13,21,25
CWD	1978	2.50	R	8,12,21					

#### Trend and Periodic Variation of Extreme Temperature Indices

**FIGURE 4:**
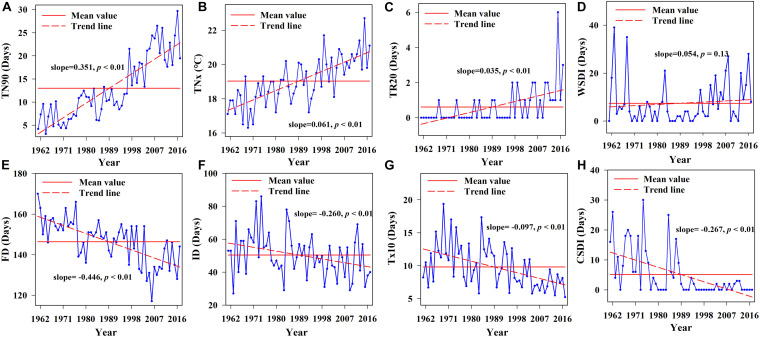
Linear trend charts of annual extreme temperature indices during 1961–2017. **(A)** linear trend chart of warm nights, **(B)** linear trend chart of monthly maximum value of min temperature, **(C)** linear trend chart of hot nights, **(D)** linear trend chart of warm spell duration indicator, **(E)** linear trend chart of frost days, **(F)** linear trend chart of icing days, **(G)** linear trend chart of cold days, and **(H)** linear trend chart of Cold spell duration indicator.

The warm indices (TN90, TNx, and TR20) significantly increased at rates of 3.51 d/10 years, 0.61°C/10 years, and 0.35 d/10 years, respectively (*p* < 0.01, Figures 4A–C). After 1989, most of the values of these indices were higher than the average values, reaching maximum values in 2016, 2015, and 2015, respectively, while WSDI showed an insignificant increasing trend. Compared with the warm indices, CSDI showed a significant decreasing trend at a rate of 2.67 d/10 years (*p* < 0.01). After 1989, the number of cold duration days was lower than average, and most cold duration days were 0 d (Figure 4D). Judging from the abrupt-changes test results, except for WSDI and FD which had extremely significant abrupt-changes in 2000, the other temperature indices had extremely significant abrupt-changes in the late 1980s and mid-to-late 1990s (Table 1). The periodic changes of extreme temperature indices were insignificant, and all extreme temperature indices did not have a consistent significant period (Table 1). Overall, the climate in the study area showed a significant warming trend after the late 1980s.

### Tree-Ring Chronology in Two Regions

**FIGURE 5:**
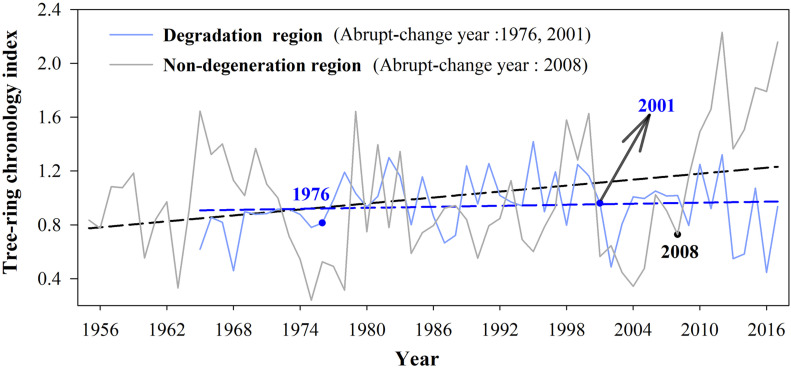
Tendency of tree-ring chronology indices in degraded regions and non-degraded region.

The tree-ring chronology showed an increasing trend in the non-degraded but a decreasing trend in the degraded regions (Figure 5). The abrupt-change year of tree-ring chronology in non-degraded region is 2008, in the degraded region is 1976 and 2001. Table 2 shows the results of trend and periodic changes of the standardized tree-ring chronology of *M. sieversii* in the two regions. The average tree-ring chronology index of *M. sieversii* was higher in the non-degraded region than the degraded region. The trend test showed an insignificantly decreasing trend of tree-ring chronology in the degraded and insignificantly increasing trend in non-degraded region (| *Z*_*c*_| < 1.98, *p* > 0.05). The tree-ring chronology in degraded region showed a significant abrupt-changes trend in 1976 (*p* < 0.01), and an insignificant abrupt-changes in 2001. The tree-ring chronology in the non-degraded region showed a very significant increase abrupt-change in 2008 (*p* < 0.01). The results of wavelet variance showed that *M. sieversii* in the non-degraded region had periods of 7, 13, and 21 years, and in the degraded region also had significant periods of 7, 12, and 21 years. The interdecadal cycles of the tree-ring chronology in the two regions were also inconsistent, with the main cycles being 12 and 13 years, respectively.

**TABLE 2:** Statistics of tree-ring chronology.

Site	Mann–Kendall trend test				Mann–Whitney test			Period
	Mean-value	*Z*_*c*_	H0	Trend	Abrupt	|*Z*_*c*_|	H0	
Degraded region	0.94	–1.37	A	No significant trend	1976	2.9	R	7,12,21
					2001	0.35	A	
Non-degraded region	1.01	1.64	A	No significant trend	2008	4.26	R	7,13,21

### Relationship Between Extreme Climate Indices and *Malus sieversii* Growth

**FIGURE 6:**
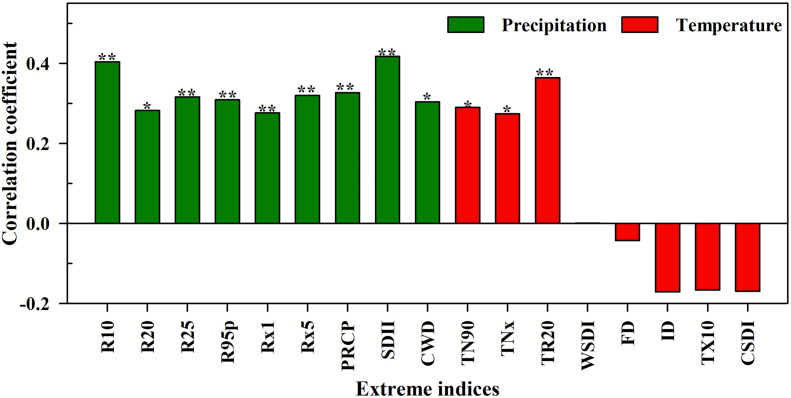
Correlations between extreme climate indices and tree-ring chronology index of *Malus sieversii* in the non-degraded area (*p < 0.05, **p < 0.01).

In arid areas, trees are more sensitive to extreme climates, and climate change has a significant impact on phenology of local vegetation. The interannual correlation coefficients of the tree-ring chronology in non-degraded areas and extreme climate indices are shown in Figure 6. Among them, R10, R25, R95P, Rx1, Rx5, PRCP, SDII, and TR20 were significantly positively correlated with the tree-ring chronology index (*p* < 0.01), and R20, CWD, TN90 and TNx were significantly positively correlated with the tree-ring chronology index (*p* < 0.05). The correlation between WSDI and tree-ring chronology was very low and FD, ID, TX10, and CSDI were insignificantly negatively correlated with tree-ring chronology. Due to the hysteresis and cumulative effects of tree-ring growth in response to climate change, this study further analyzed the multi-scale correlation between extreme climate indices and tree-ring chronology.

We conducted wavelet coherence analysis on nine extreme precipitation indices and three extreme temperature indices with significant correlations between extreme climate indices and tree-ring chronology in non-degraded areas. Among the extreme precipitation indices (Supplementary Figure 1), R10, SDII and PRCP had large and significant correlations with tree-ring chronology during the period of the 1980s–1990s, and there were cyclical changes of 4–8 and 12–20 years. Thus, R10, SDII, PRCP and the tree-ring chronology index showed a long-term consistent change, and the phase change direction was the same. Among them, SDII and the tree-ring chronology index also had a large-scale wavelet correlation on an interannual basis. Indices R20 and Rx1 had a small range of opposite phases around the 1970s, indicating that these two extreme climate events were not conducive to the growth of tree rings at this stage. Indices R20, R95p, and Rx1 showed a significant cyclical consistency of 4–8 years in the 1980s–1990s, and Rx5 and the tree-ring chronology index had a significant cyclical consistency of 8–12 years around the 1980s. The number of heavy rain days (R25) and the tree-ring chronology showed a cycle of <4 years in the 1980s, and at other times were insignificant. Thus, among the extreme precipitation indices, SDII, PRCP, and R25 had significant multi-scale correlations with tree-ring chronology in the non-degraded areas.

Among the extreme temperature indices (Supplementary Figure 2), the thermal indices (TN90 and TR20) and the tree-ring chronology had significant wavelet correlations in cycles of 4–8 and 8–16 years, respectively. The interdecadal changes of the former mainly occurred in the 1980s and 1990s, and the interannual changes mainly occurred after 2000. The interdecadal changes of the latter were around the 1990s, and the interannual changes were the same as the former. The cold index (TNx) and the tree-ring chronology had a significant wavelet correlation of 2–4-year periods after 2000, and the interdecadal changes were not significant. In general, under the interannual and interdecadal cycles, the cycle consistency between the thermal index and tree-ring chronology was better than for the cold index and tree-ring chronology.

## Discussion

### Effect of Atmospheric Circulation on Extreme Climate Change

**FIGURE 7:**
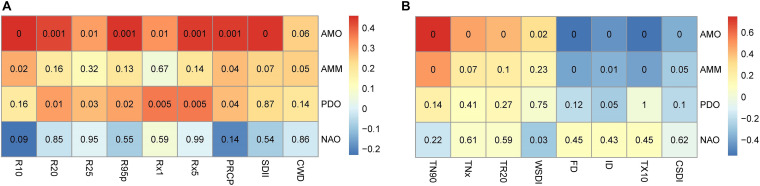
Correlation coefficients (shown in color shading) between atmospheric circulation and extreme climate indices for 1961–2017: **(A)** extreme precipitation and **(B)** extreme temperature (cell numbers are *p*-values).

Arid regions in northwest China are sensitive to global warming, and extreme climates occur frequently (Han et al., 2020; Pi et al., 2020; Xie et al., 2020). The extreme warm index in the study area increased significantly, while the extreme cold index decreased in the past 50 years (Figure 4). Due to the greater warming in winter than in summer, the increase rate of TN90 was the highest. As an important climate factor in the transition from warm and dry to warm and wet in the arid region of northwest China (Zhou and Wu, 2015; Li et al., 2016), all extreme precipitation indices showed a very significant increase trend. These changes were consistent with the trend of extreme climate change throughout the northwest (Wang B. L. et al., 2013). We also found that heavy precipitation increased by 14.04 mm/10 years, while annual total precipitation in the study area increased by 19.32 mm/10 years (Figure 3), indicating that extreme precipitation was the main factor affecting the annual total precipitation change. The trend of CWD was consistent with that of PRCP, indicating that extreme precipitation events were more frequent and serious (Wang et al., 2019). In addition, extreme precipitation indices were positively correlated with AMO, AMM and PDO, but insignificantly negatively correlated with NAO (Figure 7A). The extreme warming indices were positively correlated with AMO, AMM, and PDO, while WSDI and TN90 were negatively correlated with NAO. In contrast, the extreme cold indices were negatively correlated with AMO, AMM, and PDO, but insignificantly positively correlated with NAO (Figure 7B). Thus, AMO, AMM, and PDO had a significant influence on extreme climate change in the study area, and the extreme climate mainly was associated with AMO, consistent with the relationship between climate change and atmospheric circulation in the whole arid area of northwest China (Chen et al., 2017; Sun et al., 2020).

**FIGURE 8:**
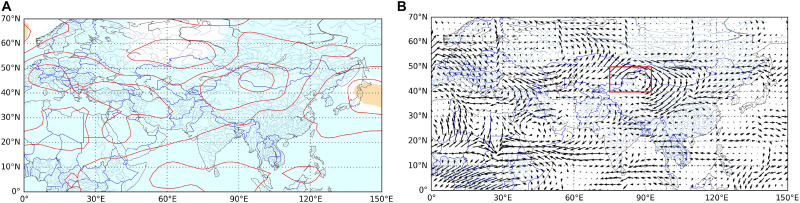
Trends of **(A)** geopotential height and **(B)** wind speed at 500 hPa during 1961–2017 (blue, *p* < 0.01; white, 0.01 < *p* < 0.05; yellow, *p* > 0.05).

Therefore, change in atmospheric circulation had a significant effect on regional extreme climate change (Shi et al., 2018). The climate warming amplitude was positively correlated with geopotential height at 500 hPa (Tang et al., 2020), and water vapor transport was susceptible to the influence of atmospheric circulation anomalies, for which the variation characteristics of geopotential height field and wind field at 500 hPa in the study area were analyzed. Geopotential height at 500 hPa in the study area had an extremely significant increasing trend, positive anomalies of potential height corresponded to higher temperature and the regional climate clearly showed a trend of increasing extreme warm indices and reducing cold indices (Figure 8A). According to the wind field at 500 hPa (Figure 8B), a cyclone formed in the south of the Qinghai-Tibet Plateau, and the study area is located in the west of the cyclone. The cyclone strengthened the south wind and promoted water vapor from the north Indian Ocean entered the arid area of northwest China, which was conducive to formation of precipitation. Additionally, there was an anti-cyclone in the southwest direction of Siberia, which promoted the occurrence of extreme precipitation in the study area by strengthening the westerly wind and bringing water vapor from the Arctic Ocean into the study area. The characteristics of geopotential height field and wind field at 500 hPa also indicated that extreme climate change was most strongly related to the AMO. In general, the influence of atmospheric circulation on precipitation and temperature in northwest China was relatively complex. For a better understanding of the relationship between regional extreme climate change and atmospheric circulation, it is particularly important to analyze their changes and relationships using multi-scales in future studies.

### Effect of Extreme Climate Change on Tree-Ring Growth

The tree-ring chronology of *M. sieversii* in non-degraded areas showed an increasing trend (Figure 5), which had a positive relationship with extreme precipitation indices (Figure 6). There was a significant correlation and consistent change in the 1980s–1990s of a 4–8-year period (Supplementary Figure 1), indicating that a consistently wet climate increased the radial growth of trees in forests in this arid region. Extreme precipitation promotes the growth of trees. On one hand, sufficient precipitation can promote tree growth by accelerating photosynthesis and carbon storage of tree to improve the effectiveness of trees in the utilization of hydrothermal resources (Sala et al., 2012; Jiang et al., 2019). On the other hand, increasing precipitation can increase decomposition of litter by microorganisms, promoting the growth of trees by improving the effectiveness of nitrogen (Sala et al., 2012). The tree-ring chronology in the non-degraded area was positively correlated with the extreme temperature index and the warm index (TN90, TNx, and TR20), and showed good consistency in 8–16-year period change during the 1980s–1990s. This is because warmer temperatures can close stomates and promote carbon storage in trees, and warmer spring temperatures can promote leaf initiation and tree-ring formation. The positive correlation between tree-ring chronology and WSDI was relatively small in the non-degraded area, possibly due to the adaptability of trees to long-term environmental changes (Drake et al., 2010; Reich et al., 2016). Our study showed that the effect of extreme temperature index was smaller than that of extreme precipitation, and tree-ring chronology was the most sensitive to the change of SDII according to the correlation coefficient values. This was mainly because the study area was located in the arid region of northwest China (Zhou and Wu, 2015; Li et al., 2016). In recent years, the climate has tended to be humid and water resources are the main factors affecting the local vegetation growth. Although temperature in the study area increased year by year and extremely high temperatures and other events were not conducive to tree growth, the fertile soil, and abundant precipitation reversed the negative effect of the extreme high temperature events on tree growth (Scharnweber et al., 2020).

**FIGURE 9:**
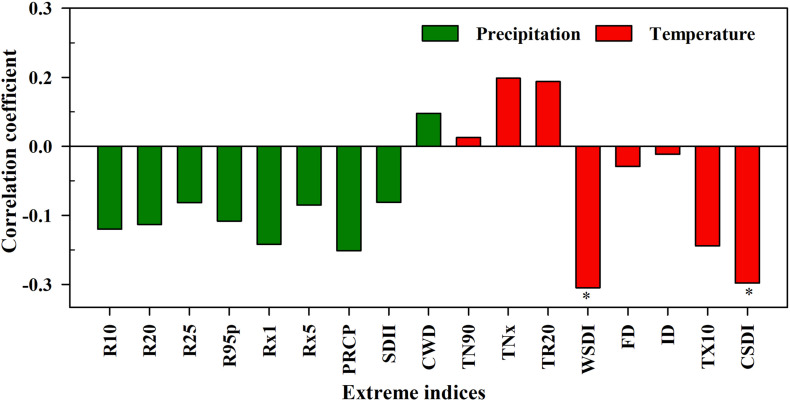
Correlation coefficients of extreme climate indices and the tree-ring chronology of *Malus sieversii* in the degraded region.

However, the tree-ring chronology of *M. sieversii* in the degraded area was decreasing (Figure 5), under insignificant difference between climatic conditions and soil conditions in the two regions (Supplementary Table 2). Combining investigation by managers of *M. sieversii* forest with research (Liu et al., 2014; Chen, 2015; Fang et al., 2019), the main difference between the two areas is due to the different levels of human disturbance. The *M. sieversii* forests have experienced minor destruction in the non-degraded region. In contrast, local people began to develop and utilize wild fruit forest resources since 1970. A large number of wild apples were gathered and some trees were cut down by local people in 1970. Local people realized the importance of protecting the *M. sieversii* forests and started to appropriately gather wild apples for making wine since 1980. Since 1990, the utilization of wild apple forest resources has reduced, the protection was strengthened by local inhabitants. In 1995, the new cultivated apple species from China’s Shandong Province were introduced into the degraded region, which caused the first detection of *Agrilus mali Matsumura* insects in wild apple trees (Chen, 2015). Local measures of perforating and injecting chemicals to control pests, but the *Valsa ceratosperma* occurred in trees. Local government departments also protected the trees by cutting off diseased branches, and insect control was also carried out through flying control since 2000. Our multi-scale correlation analysis between extreme climatic indexes and tree-ring chronology in the degraded region showed that tree-ring chronology had significant negative correlations with WSDI and CSDI (Figure 9). However, the wavelet correlation coefficients between tree-ring chronology and extreme precipitation indices (Supplementary Figure 3) showed that in the degraded region, R10, R95p, CWD, SDII, and PRCP were significantly correlated with tree-ring chronology in a 4–8-year period, and tree-ring chronology was significantly correlated with WSDI and CSDI in a 2–8-year periods (Supplementary Figure 4).

The main reasons are as follows. The measures of perforating and injecting chemicals in the early growing season (around April) to control pests promoted the occurrence of *V. ceratosperma*, following extreme precipitation events (mainly in June), which impeded growth of wild apple trees. Extreme high temperature events occur frequently in July in the study region, and are conducive to outbreaks of diseases and insect pests. Human measures of sawing branches further reduced the leaf area of *M. sieversii* trees, resulting in the reduction of photosynthetic production, which hindered the growth of *M. sieversii* trees in the area (Molina et al., 2019). Overall, the occurrence of extreme precipitation and temperature promoted the growth of wild apple tree rings in the degraded area. Moreover, due to unreasonable human activities, such as deforestation, agricultural reclamation and grazing, *M. sieversii* is more vulnerable to climate change, which is not conducive to recovery of *M. sieversii* growth following extreme climate events. As a consequence, the growth of damaged trees would further deteriorate, leading to further degradation of *M. sieversii* forest. Under the conditions of increasing extreme precipitation and extreme warm climate events, growth of trees in the non-degraded area was better, but was worse in the degraded area. These results showed that both unreasonable anthropogenic activities and extreme climate caused outbreaks of pests and diseases, and resulted in degeneration of wild fruit forest. Most studies have focused on the impact of environmental and climate stress (including extreme climate events) on tree growth, while ignoring the human impacts on management and disturbance of tree growth in forests (Julio Camarero et al., 2011; Sidor et al., 2019). In recent years, many trees have been negative growth and even widespread death in the world (Gazol et al., 2015, 2018; Martinez del Castillo et al., 2019; Decuyper et al., 2020). It is necessary to analyze the reasons for changes in tree growth resulting from combined the effects of anthropogenic activities and climate change in future studies.

### Ecological Conservation Measures to Adapt to Extreme Climate Change

In dry forest, decayed individuals are able to achieve their pre-drought growth levels following drought, while non-decayed individuals are able to improve further following drought, showing that trees have strong resistance and resilience to short-term drought (Julio Camarero et al., 2018). *M. sieversii* is the ancestor of cultivated apple but has greater ability to resist environmental stress (Wuerdig et al., 2015; Yang et al., 2017, 2019; Wang et al., 2018). Because of their excellent resistance, the vitality of good tree individuals can be strengthened, with poor trees suffering from human disturbance can become worse. For example, improper pruning by humans reduces the water use efficiency of trees (Molina et al., 2019), extreme high temperature exacerbates insect and disease outbreaks, and extreme precipitation can cause outbreaks of *Valsa mali* Miyabe et Yamada. These resulted in the growth of *M. sieversii* showing a decreasing trend, and the correlation between tree-ring chronology in the degraded area and extreme precipitation, and high temperature indices showed negative correlations (Supplementary Figures 3, 4 and Figure 9). Hence, it is effective to promote tree growth through appropriate pruning after extreme climate events during the growing season. During outbreaks of pests and diseases in wild fruit forests, the control of tree infection by sawing off diseased and dead branches can weaken the resistance of the trees and reduce their ability to cope with extreme climate events. Therefore, it is necessary to strengthen research on pathogenic mechanisms and phenology of *M. sieversii* to enable the use of appropriate chemical control combined with biological control technology. Following extreme weather events, the growth status of trees is related to their previous growth status, and the growth of *M. sieversii* in the non-degraded area showed an increasing trend. Therefore, it is important to strengthen the monitoring of individual tree growth for the timely detection of growth problems and the development of solutions (Hartmann et al., 2018). It is also important to focus on the protection of poor trees in future forest protection and management. In addition, wild fruit forests are world class wild resource banks. In the process of degradation, many wild species become endangered (Liu et al., 2014). Therefore, the establishment of gene banks for wild resources should be continuously strengthened.

## Conclusion

In the past 50 years, both the extreme precipitation and extreme warm indices showed an increasing trend while extreme cool indices showed a decreasing trend in the study area. The extreme precipitation indices and extreme warm indices in the study area were positively correlated with the large-scale circulation indices of AMO, AMM and PDO, and negatively correlated with NAO. The geopotential height field and wind field at 500 hPa in the study area showed increasing trends. Changes in both the Siberian anti-cyclone and the Qinghai-Tibet Plateau cyclone near the study area resulted in changes in extreme climate events jointly.

Among the extreme climate indices, the number of days in which extreme cold events occurred tended to decrease in recent years and their effect on tree-ring chronology weakened. Extreme precipitation indices and extreme warmth indices (TN90, TNx, and TR20) showed significantly increasing trends, and were significantly positively correlated with the tree-ring chronology, indicating promoted growth in *M. sieversii* of the non-degraded forest. There were common periods of 12–13 years between tree-ring chronology and both the extreme precipitation, and extreme warm indices, indicating extreme climate events promoted the periodic change in tree-ring chronology of *M. sieversii* in the non-degraded region.

Compared with the increasing growth trend of the non-degraded trees, *M. sieversii* growth in the degraded area showed an insignificant decreasing trend. Pearson’s correlation coefficients showed insignificant negative correlations between tree-ring chronology and R10, R95p, CWD, SDII, and PRCP. However, there were significant correlations with tree-ring chronology in a 4–8-year period. Tree-ring chronology in degraded areas was significantly negatively correlated with WSDI and CSDI, and significantly correlated with CSDI in a 2–8-year period. Extreme precipitation and WSDI had a significantly negative effect on the wild fruit forest over a long time-scale. In addition, because of inappropriate anthropogenic disturbance, extreme climate change further aggravated the frequent occurrence of pests and diseases, leading to the degradation of wild fruit forests.

## Data Availability Statement

The raw data supporting the conclusions of this article will be made available by the authors, without undue reservation.

## Author Contributions

QS achieved the analysis of the climate change and tree growth trends, the computation of dendrochronological/climate correlations, and the writing – original manuscript. HL provided the framework, conceptualization, and methodology. HZ and QS calculated the extreme climate index and visualized all results. ML and ZW finished the measurement of tree-ring width, the tree-ring chronology established, and participated in the investigation. GZ finished the formal analysis, and provided conceptualization and methodology. All authors contributed to the article and approved the submitted version.

## Conflict of Interest

The authors declare that the research was conducted in the absence of any commercial or financial relationships that could be construed as a potential conflict of interest.

## Footnotes

1 https://www.esrl.noaa.gov/

2 https://psl.noaa.gov/data/gridded/data.ncep.reanalysis.pressure.html

3 http://etccdi.pacificclimate.org/software.shtml

4 http://etccdi.pacificclimate.org/
