# Supplementary material for: Do Extreme Climate Events Cause the Degradation of Malus sieversii Forests in China?
Source: Front Plant Sci. 2021 Jun 16;12:608211. doi: 10.3389/fpls.2021.608211 (PMC8244594; doi:10.3389/fpls.2021.608211)
Supplement: Supplementary file 1 [file Data_Sheet_1.docx]

***Supplementary Material***

1. **Supplementary Figures and Tables**

**1.1 Supplementary Figures**


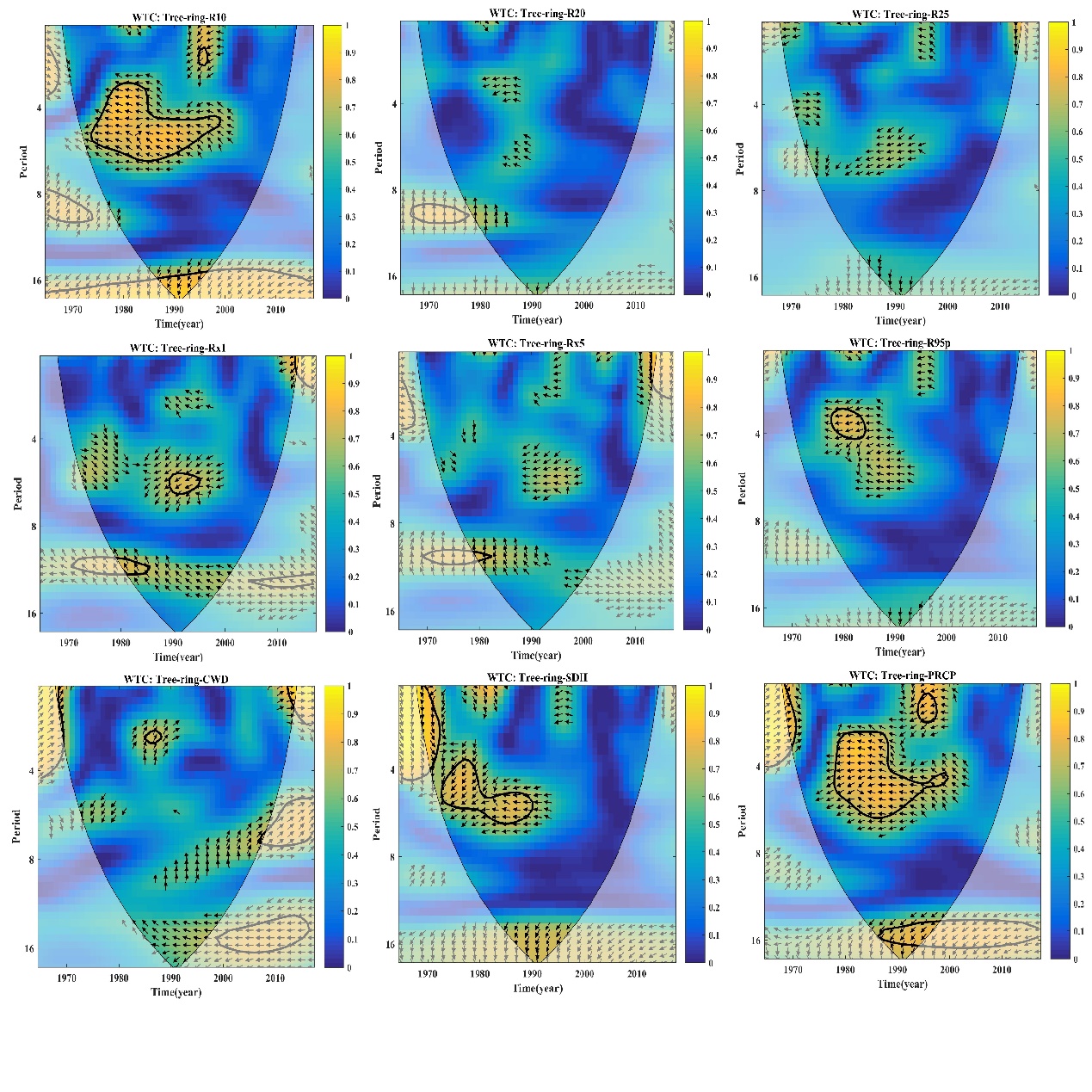


**Supplementary Figure 1. Wavelet coherence spectrum of extreme precipitation index and tree-ring chronology index in the non-degraded region**


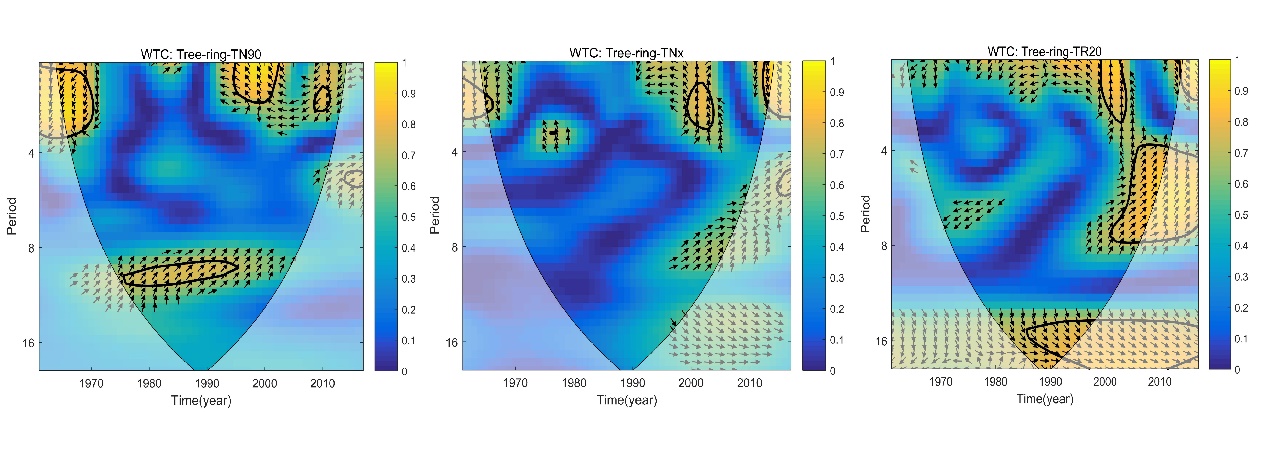


**Supplementary Figure 2.** **Wavelet coherence spectrum of extreme temperature index and tree-ring chronology index** **in the non-degraded region**


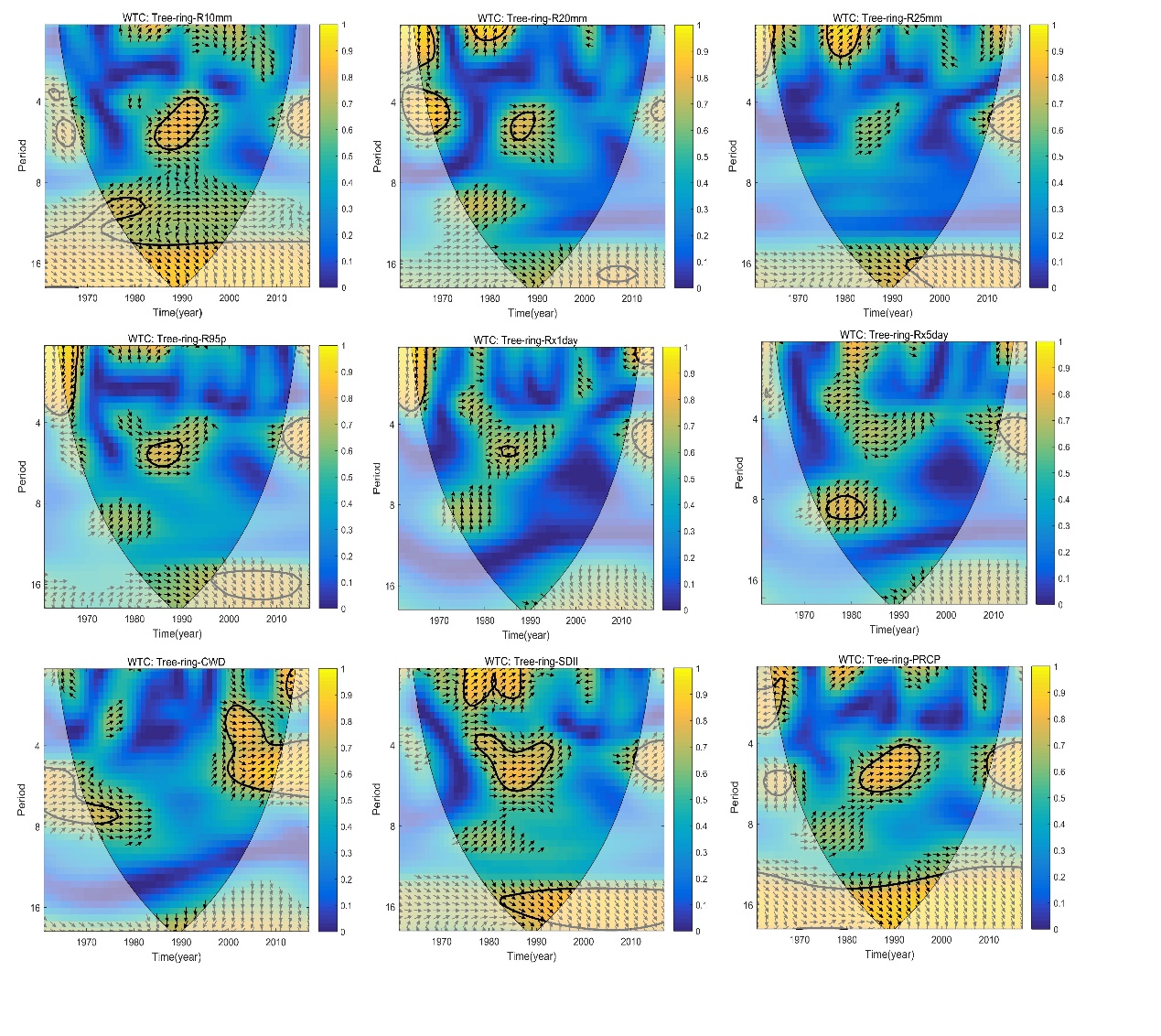


**Supplementary Figure 3. The wavelet coherence spectrum between tree-ring chronology and extreme precipitation indices in the degraded region**


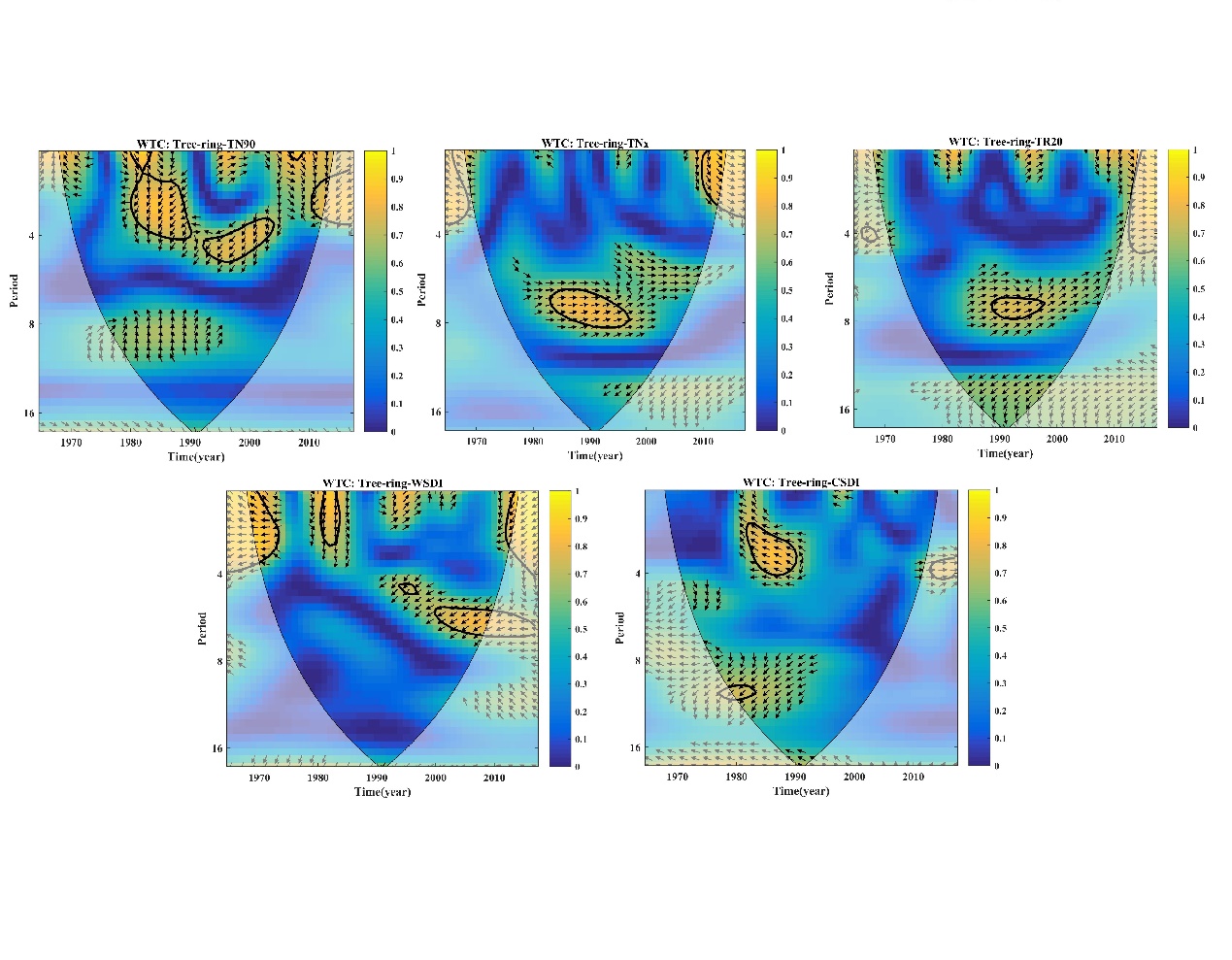
**Supplementary Figure 4. The wavelet coherence spectrum between tree-ring chronology and extreme temperature indices in the degraded region**

**1.2 Supplementary Tables**

**Supplementary Table 1.** Degenerated grading standards of study state

| Degenerated levels | Grading standards |
| --- | --- |
| Non-degenerated region | The crown has no or minor defect, or deadwood accounts for less than 1/3 of the total, leaves are dark or grey green, and the crown loss is 10%~40%. |
| Degenerated region | The crown has larger defect, deadwood accounts 1/3~3/4 of the total, leaves are grey green or a few leaves exist, and the crown loss is between 40%~80% or more than 80%. |

**Supplementary Table 2.** Results of soil difference test among two regions

| Item | Study state | pH | TS (mg/g) | OC（g/kg） | T N（g/kg) | OM（g/kg） |
| --- | --- | --- | --- | --- | --- | --- |
| Mean | Non-degenerated region | 7.57 | 1.55 | 65.45 | 6.50 | 112.83 |
|  | Degenerated region | 7.18 | 0.84 | 66.00 | 7.04 | 113.78 |
| Variance | Non-degenerated region | 0.38 | 1.46 | 602.73 | 5.13 | 1791.42 |
|  | Degenerated region | 0.15 | 0.36 | 667.35 | 1.70 | 1983.44 |
| ANOVA | F | 4.49 | 4.49 | 4.49 | 4.49 | 4.49 |
|  | p-value | 0.12 | 0.13 | 0.96 | 0.54 | 0.96 |

TS, total salt; OC, organic carbon; TN, total nitrogen; OM, organic matter.

**Supplementary Table 3.** Chronology statistics of two regions' wild fruit tree

| Sampling sites | Period | AC1 | SNR | SD | Rbar | EPS |
| --- | --- | --- | --- | --- | --- | --- |
| Non-degraded region | 1955~2017 | 0.48 | 3.45 | 0.08 | 0.54 | 0.78 |
| Degraded region | 1965~2017 | 0.15 | 2.45 | 0.13 | 0.26 | 0.72 |

AC1, first-order autocorrelation; MS, mean sensitivity; SNR, signal to noise ratio; SD, standard deviation, Rbar, mean inter-series correlation; EPS, expressed population

**Supplementary Table 4. Definition of all extreme indices in the study**

| \| ID \| Name \| Description \| Unit \| \| --- \| --- \| --- \| --- \| \| Precipitation \|  \|  \|  \| \| RX1, RX5 \| Maximum one-day precipitation; Maximum five-day precipitation \| Highest precipitation amount in one-day period; Highest precipitation amount in five-day period \| mm \| \| R10, R20, R25 \| Light rain, Moderate rain, heavy rain \| Annual count of days when annual total precipitation ≥ 10mm, 20mm, 25mm \| Days \| \| R95p \| Very wet days \| Annual total precipitation when daily total precipitation > 95th percentile \| mm \| \| PRCP \| Annual total precipitation \| The sum of the daily precipitation of all the rain (snow) in a year \| mm \| \| SDII \| Simple precipitation intensity index \| The ratio of the total precipitation with daily precipitation ≥1.0mm to the number of precipitation days \| mm/d \| \| CWD \| Consecutive wet days \| Maximum consecutive days with daily precipitation ≥1.0mm \| Days \| \| Temperature \|  \|  \|  \| \| TX10 \| Cold days \| Number of days when daily max temperature < 1961-2017 10th percentile \| Days \| \| TN90 \| Warm nights \| Number of days when daily min temperature > 1961-2017 90th percentile \| Days \| \| TR20 \| Hot nights \| The number of days in which the daily minimum temperature is greater than 20 ℃ \| Days \| \| TNx \| Max Tmin \| Monthly maximum value of min temperature \| ℃ \| \| ID、FD \| Number of icing days; Number of frost days \| Annual count when max temperature, min temperature < 0℃ \| Days \| \| CSDI \| Cold spell duration indicator \| Annual count of days with at least 6 consecutive days when min temperature < 10th percentile \| Days \| \| WSDI \| Warm spell duration indicator \| Annual count of days with at least 6 consecutive days when max temperature > 90th percentile \| Days \| |
| --- | --- | --- | --- | --- | --- | --- | --- | --- | --- | --- | --- | --- | --- | --- | --- | --- | --- | --- | --- | --- | --- | --- | --- | --- | --- | --- | --- | --- | --- | --- | --- | --- | --- | --- | --- | --- | --- | --- | --- | --- | --- | --- | --- | --- | --- | --- | --- | --- | --- | --- | --- | --- | --- | --- | --- | --- | --- | --- | --- | --- | --- | --- | --- | --- |
